# Diffusion control of an ion by another in LiNbO_3_ and LiTaO_3_ crystals

**DOI:** 10.1038/srep10018

**Published:** 2015-05-05

**Authors:** De-Long Zhang, Qun Zhang, Cong-Xian Qiu, Wing-Han Wong, Dao-Yin Yu, Edwin Yue-Bun Pun

**Affiliations:** 1 Department of Opto-electronics and Information Engineering, School of Precision Instruments and Opto-electronics Engineering, Tianjin University, Tianjin 300072, People’s Republic of China; 2 Key Laboratory of Optoelectronic Information Technology (Ministry of Education), Tianjin University, Tianjin, 300072, People’s Republic of China; 3 Department of Electronic Engineering and State Key Laboratory of Millimeter Waves, City University of Hong Kong, 83 Tat Chee Avenue, Kowloon, Hong Kong, People’s Republic of China

## Abstract

Diffusion-doping is an effective, practical method to improve material properties and widen material application. Here, we demonstrate a new physical phenomenon: diffusion control of an ion by another in LiNbO3 and LiTaO3 crystals. We exemplify Ti^4+^/X^n+^ (X^n+^ = Sc^3+^, Zr^4+^, Er^3+^) co-diffusion in the widely studied LiNbO3 and LiTaO3 crystals. Some Ti^4+^/X^n+^-co-doped LiNbO3 and LiTaO3 plates were prepared by co-diffusion of stacked Ti-metal and Er-metal (Sc2O3 or ZrO2) films coated onto LiNbO3 or LiTaO3 substrates. The Ti^4+^/X^n+^-co-diffusion characteristics were studied by secondary ion mass spectrometry. In the X^n+^-only diffusion case, the X^n+^ diffuses considerably slower than the Ti^4+^. In the Ti^4+^/X^n+^ co-diffusion case, the faster Ti^4+^ controls the diffusion of the slower X^n+^. The X^n+^ diffusivity increases linearly with the initial Ti-metal thickness and the increase depends on the X^n+^ species. The phenomenon is ascribed to the generation of additional defects induced by the diffusion of faster Ti^4+^ ions, which favors and assists the subsequent diffusion of slower X^n+^ ion. For the diffusion system studied here, it can be utilized to substantially shorten device fabrication period, improve device performance and produce new materials.

Doping by thermal diffusion is an effective and practical method for improving material properties and widening material application. Here, we exemplify Ti^4+^/X^n+^ (X^n+^ = Sc^3+^, Zr^4+^, Er^3+^) co-diffusion in LiNbO_3_ (LN) and LiTaO_3_ (LT) crystals to demonstrate an interesting phenomenon that a faster ion controls diffusion of another slower ion in the two crystals.

Er^3+^-doped LN crystal is a promising material for integrated optics as it combines excellent electro-optic, acousto-optic and nonlinear optical properties of LN with good laser property of Er^3+^. Such an effective combination, together with the possibility of producing a high-quality waveguide, enables broadband amplification and lasing in the telecom wavelength region. Over the past years, a family of Ti (or vapor ZnO)-diffused Er:LN waveguide lasers (amplifiers) and integrated devices have been demonstrated^1^−^3^. For any Ti:Er:LN device, selective Er^3+^ doping is a prerequisite for monolithic integration of active (optically pumped, Er^3+^-doped) and passive (unpumped) devices on a same substrate, to avoid re-absorption in unpumped Er^3+^-doped waveguide. The doping is realized by in-diffusion of Er metal or its oxide at a temperature close to the Curie point of crystal. Previous study shows that Er^3+^ solubility in LN is limited and the diffusivity is rather low^4^,^5^. Very low diffusivity results in very long diffusion time of >100 h. Moreover, the serious photorefractive effect in the LN not only affects the device performance, but also limits both the pumping and operating wavelengths and hence hinders further development of novel devices. It is well known that doping with >4.9 mol% MgO can effectively suppress the effect^6^. However, as the crystal is heavily MgO-doped, both the diffusivity and solubility degrade further^7^. This is undesired as the optical gain of a laser or an amplifier increases with the active ion concentration and the low diffusivity results in quite long device fabrication period and hence increased cost. It is imperative to seek other dopants that have a low threshold concentration of photorefractive damage. In addition to the much studied Mg^2+^ 4, other dopants can also effectively suppress the photorefractive effect. These include divalent Zn^2+^ 8, trivalent Sc^3+^ 9,10, In^3+^ 11 and Tm^3+^ 12, and tetravalent Hf^4+^ 13, Zr^4+^ 14, and Sn^4+^ 15. Among them, the Sc^3+^ and Zr^4+^ show the lower concentration threshold of photorefractive damage, only 2 mol%. The low Sc^3+^ or Zr^4+^ doping concentration enables to increase the Er^3+^ solubility and diffusivity and improve the material homogeneity for nonlinear use as well. Thus, an LN doped with >2 mol% Sc^3+^ or Zr^4+^ would be a more promising material than the Mg-doped one for developing an optical-damage-resistant device.

A Ti-diffused LN (Ti:LN) waveguide is a basic component of an LN-based waveguide device. As an alternative, a Ti:LN waveguide co-doped with Zr^4+^ or Sc^3+^ can be fabricated by Ti^4+^ diffusion following Zr^4+^ or Sc^3+^-doping. And a Ti:Er:LN co-doped with Zr^4+^ or Sc^3+^ can be fabricated by successive diffusion of Er^3+^, Zr^4+^ (or Sc^3+^) and Ti^4+^. For these two kinds of waveguides, either Ti^4+^/Er^3+^, Ti^4+^/Zr^4+^ or Ti^4+^/Sc^3+^ co-diffusion is concerned in the Ti^4+^ diffusion procedure. Here we show that the Ti^4+^ diffuses considerably faster than the X^n+^ ( = Er^3+^, Zr^4+^or Sc^3+^) and can control the X^n+^ diffusion, enabling to shorten the device fabrication period, lower the cost, promote the Er^3+^ concentration and improve the device performance.

In comparison with LN, the LT crystal has similar crystal and defect structures, and similar electro-optic and nonlinear properties. Moreover, the LT shows two orders of magnitude higher resistance to the photorefractive damage and has the higher melting temperature of 1650 °C favorable for impurity doping by diffusion method. The LT may find applications similar to the LN. The co-diffusion study is also carried out on the LT crystal.

Fig. 1 shows the measured depth profiles of ^6^Li, ^93^Nb, ^16^O, ^45^Sc and/or ^48^Ti SIMS signals detected from the LN plates coated with (a) 160 nm Ti or 50 nm Sc_2_O_3_, (b) 50 nm Sc_2_O_3_ + 50 nm Ti, (c) 70 nm Sc_2_O_3_ + 115 nm Ti, and (d) 160 nm Ti + 118 nm Sc_2_O_3_ films after annealing at 1060 ^o^C for 10 h in wet O_2_. On each panel, the red ball curve represents the measured Sc^3+^ profile and the magenta ball curve denotes the measured Ti^4+^ profile. In order to save space, Fig. 1(a) simultaneously shows the two cases of Sc^3+^- and Ti^4+^-only diffusion. Because the substrate signal profiles are similar for the two cases of Sc^3+^- and Ti^4+^-only diffusion, here only the substrate signals detected from the Sc^3+^-only diffused sample are shown in Fig. 1(a). We note that all of the substrate signals ^6^Li, ^93^Nb and ^16^O show constancy with depth during the analysis. This is expected because the concentration profiles of these substrate constituents should be homogeneous over the crystal plates. It is found that all of the measured Sc^3+^ and Ti^4+^ profiles shown in Fig. 1 can be well fitted by a Gaussian function, whether it is the single diffusion or co-diffusion,
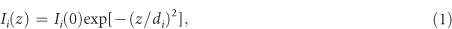


where *I*_*i*_(z) (*i* = Sc or Ti) represents the yield of secondary Sc^3+^ or Ti^4+^ ions, *d*_*i*_ denotes the 1/e Sc^3+^ or Ti^4+^ diffusion depth. The fitting results are plotted by the green line for Sc^3+^ and blue for Ti^4+^. The fitting expression is indicated for each case. One can see that there is an excellent agreement between the fitting and measured curves for each sample, showing that the diffusion reservoir has been exhausted for all of the studied Ti/Sc_2_O_3_ samples. In the single diffusion case, *d*_Sc_ (*d*_Ti_) = 3.8 (7.0) ± 0.2 μm, yielding a diffusivity of (0.26 ± 0.03) [1.23 ± 0.07] μm^2^/h for Sc^3+^[Ti^4+^]. In the co-diffusion case, the *d*_Sc_ (*d*_Ti_) value is 4.4 (6.3), 5.3 (6.4), 5.5 (6.5), 5.8 (6.4) and 6.4 (6.6) ± 0.2 μm for the Ti/Sc_2_O_3_ samples 3–7, respectively. The diffusivity *D*_Sc_ [*D*_Ti_] is 0.48 ± 0.04 [0.99 ± 0.06], 0.70 ± 0.05 [1.02 ± 0.06] 0.75 ± 0.06 [1.06 ± 0.07], (0.84 ± 0.06) [1.02 ± 0.06] and 1.02 ± 0.06 [1.09 ± 0.07] μm^2^/h, respectively.

Fig. 2 shows the depth profiles of ^7^Li, ^109^NbO, ^32^O_2_, ^123^ZrO_2_ and/or ^64^TiO negative-ion SIMS signals detected from five representative LN samples that were initially coated with (a) 160 nm Ti or 80 nm Zr_2_O_2_, (b) 60 nm ZrO_2_ + 65 nm Ti, (c) 60 nm ZrO_2_ + 135 nm Ti, and (d) 60 nm ZrO_2_ + 185 nm Ti films. Fig. 2(a) shows the Zr^4+^-only diffusion case. For convenience, the Ti^4+^-only diffusion profile is again included in Fig. 2(a). Similar to the Ti^4+^/Sc^3+^ co-diffusion case, both the Ti^4+^ and Zr^4+^ profiles follow well the Gaussian function too. The fitting expression is indicated for each case. In the single diffusion case, *d*_Zr_ = 3.4 ± 0.2 μm, yielding a diffusivity of (0.29 ± 0.04) μm^2^/h. In the co-diffusion case the *d*_Zr_ (*d*_Ti_) is 4.0 (6.8), 4.4 (6.9), 4.7 (7.1) and 5.0 (7.0) ± 0.2 μm for the Ti/ZrO_2_ samples 3, 4, 5 and 6, respectively. The resultant diffusivity *D*_Zr_ [*D*_Ti_] is 0.40 ± 0.04 [1.16 ± 0.07], 0.48 ± 0.04 [1.19 ± 0.07], 0.55 ± 0.05 [1.26 ± 0.07] and (0.63 ± 0.05) [1.23 ± 0.07] μm^2^/h, respectively.

For the Ti/Er LN samples, for which the SIMS results are not shown for save space, under the diffusion condition adopted, 1130 ^o^C/50 h, the Ti^4+^ ion penetrates too deep to attain its entire profile using the SIMS technique. But the entire Er^3+^ profile, which we are more interested in, can be obtained. Analysis shows that the measured Er^3+^ profile follows also the Gaussian function for all of the studied Ti/Er LN samples. The fit yields a *d*_Er_ value of 3.6, 4.4, 4.8, 5.0, 5.6, 6.2 and 4.7 ± 0.2 μm for the Ti/Er LN samples 1–7, respectively. The resulting diffusivity *D*_Er_ is 6.5 ± 0.06, 9.7 ± 0.09, 11.8 ± 1.1, 12.6 ± 1.2, 15.7 ± 1.4, 19.2 ± 1.5 and (11.1 ± 1.0) × 10^−2^ μm^2^/h, respectively.

In the following we discuss the Ti^4+^/X^n+^ co-diffusion features and the mutual influence issue of Ti^4+^ and X^n+^ diffusion in the LN. First, we compare the diffusivities of the related four ions in the single diffusion case. The Ti^4+^ diffusion in the LN crystal has been studied since the 1980’s 16,17,18,19, and the diffusivity data reported show a large diversity perhaps due to different extents of suppression for Li_2_O out diffusion. The Ti^4+^-only diffusivity here, ~1.23 μm^2^/h @ 1060 ^o^C, is closer to the value reported by Fukuma and Noda, ~0.98 μm^2^/h^17^. A comparison shows that in the single diffusion case the Zr^4+^, Sc^3+^ and Er^3+^ ions diffuse respectively four-, five-fold and one order of magnitude slower than the Ti^4+^. The ion for the diffusivity from high to low is Ti^4+^, Zr^4+^, Sc^3+^ and Er^3+^ in order. This is associated with their differences in atomic mass, ionic radius and chemical valence as well.

Next, we focus on the Ti^4+^/X^n+^ co-diffusion features in the LN. We note from the diffusivity data given above that the Ti^4+^ ion assists and controls the diffusion of all the X^n+^ ions. The co-diffusion of Ti^4+^ leads to considerable increase in X^n+^ diffusivity and the X^n+^ diffusivity increases with the initial thickness of Ti-metal film coated. As the Ti thickness is increased from zero to ~200 nm, the X^n+^ diffusivity increases from 0.26 to 1.02 μm^2^/h for Sc^3+^, from 0.30 to 0.62 μm^2^/h for Zr^4+^, and from 6.5 × 10^−2^ to 19.2 × 10^−2^ μm^2^/h for Er^3+^. The increase is more than four-, two- and three-fold, respectively. To quantify the relation of X^n+^ diffusivity to Ti thickness, in Fig. 3 we plot the X^n+^ diffusivity against the initial Ti thickness (red balls for Sc^3+^, green balls for Zr^4+^ and magenta balls for Er^3+^). The error bar is indicated for each data. One can see that the diffusivity and the initial Ti thickness follow a linear relationship for all the X^n+^ ions. The red, green and magenta lines in Fig. 3 represent the linear fits to the respective experimental data. The fitting expression is D_Sc_ [μm^2^/h] = 0.276 + 3.72 × 10^−3^ × (Ti thickness) for Sc^3+^, D_Zr_ [μm^2^/h] = 0.295 + 1.62 × 10^–3^ × (Ti thickness) for Zr^4+^, and D_Er_ [μm^2^/h] = 0.0659 + 6.405 × 10^–4^ × (Ti thickness) for Er^3+^. The fitting error is within 10%.

In contrast, the Ti^4+^ diffusivity in the co-diffusion case changes little. For straightforwardness, the Ti^4+^ diffusivity is also plotted in Fig. 3(a). The red (green) squares represent the Ti^4+^ diffusivity in the case of co-diffusion with the Sc^3+^(Zr^4+^). The line is drawn to guide the eyes only. In the Ti^4+^/Er^3+^ co-diffusion case, the Ti^4+^ diffusivity is not available as we cannot obtain the entire Ti^4+^ profile. One can see that the co-diffusion of Sc^3+^ only induces slight decrease of Ti^4+^ diffusivity from ~1.2 to ~1 μm^2^/h and the decrease is no more than 20%. In the Ti^4+^/Zr^4+^ co-diffusion case, the Ti^4+^ diffusivity is hardly influenced by the Zr^4+^ co-diffusion. It remains unchanged within the error no matter how the initial Ti or ZrO_2_ film thickness changes, i. e., in the co-diffusion case the Ti^4+^ diffusion is actually in the scenario of single diffusion. Moreover, the Ti^4+^ diffusivity is independent of the Ti thickness (i. e. independent of the Ti^4+^ concentration), consistent with the single-diffusion case. In addition, the Ti^4+^ diffusivity is also independent of the initial Sc_2_O_3_ or ZrO_2_ film thickness.

Subsequently, we pay attention to the effect of coating sequence on the diffusion. As described above, the samples have different coating sequences of X^n+^ and Ti films (see Table 1 for details). We note from Fig. 3 that both X^n+^ and Ti^4+^ diffusivities appear not to be influenced by the coating sequence. Here we exemplify the Ti^4+^/Er^3+^ samples 3 and 7 to demonstrate it. One can see from Table 1 that the two samples were coated with the same thickness of Er (22 nm) and Ti (76 nm). Their only difference is in the coating sequence. We note that the Er^3+^ in the two samples has the similar diffusivities, (11.8 ± 1.1) × 10^–2^ μm^2^/h in sample 3 and (11.1 ± 1.0) × 10^–2^ μm^2^/h in sample 7. We may conclude that the coating sequence does not affect the Er^3+^ diffusivity. This should be also true for the Ti^4+^ diffusivity.

Similar feature that the Ti^4+^ ion assists and controls Er^3+^ diffusion is also observed for the Ti^4+^ /Er^3+^ co-diffusion system in the LT crystal. From the measured Er^3+^ profiles, which follow either a Gaussian function or an error function of complement (erfc), the Er^3+^ diffusivities at different diffusion temperatures of 1200, 1300, 1400, 1450 and 1500 °C are obtained and given in Table 2. For straightforwardness, the diffusivities are plotted against the inverse of temperature 1/T (in unit of 1/K) in Fig. 4 for both cases of Er^3+^/Ti^4+^ co-diffusion and respective single diffusion. For reader’s convenience, the temperature is indicated for each data point. The red balls represent the Er^3+^ diffusivities in the case of Er^3+^-only diffusion. The red line represents the linear fit to the red balls on the semi-logarithmic scale and the fitting expression is indicated. The blue balls denote the Er^3+^ diffusivities in the Ti^4+^/Er^3+^ co-diffusion case. The blue data indicated near the blue balls denote the initial thicknesses of Ti-metal film coated. Indeed, one can see that for a given temperature the Ti^4+^ co-diffusion leads to definite increase in Er^3+^ diffusivity. The Er^3+^ diffusivity increases with the increased initial Ti-metal film and the increase ranges from 1.6 to 6-folds, depending on the initial thickness of the Ti-metal film coated.

Like in the co-diffusion case in LN, the Ti^4+^ diffusivity in the co-diffusion case in LT changes little too. In Fig. 4 the magenta balls represent the Ti^4+^ diffusivities in the Ti^4+^/Er^3+^ co-diffusion case (For some LT samples, an entire Ti^4+^ profile can be obtained, enabling to obtain the Ti^4+^ diffusivity. This is distinguished from the LN case). The magenta line represents the temperature dependence of Ti^4+^ diffusivity in the Ti^4+^-only diffusion case. The line is drawn from the data calculated using the diffusion constant and activation energy values reported for Ti^4+^ single diffusion in LT^20^. We note that in the single diffusion case in LT the Ti^4+^ diffusivity is at least two orders of magnitude larger than the Er^3+^ diffusivity. In the co-diffusion case in LT the Ti^4+^ diffusivity does not reveal a remarkable difference from that in the Ti^4+^-only diffusion case, consistent with the case in LN. In addition, the Er^3+^ and Ti^4+^ diffusivities in the co-diffusion case in LT are also not affected by either the initial thickness of Er-metal film coated or the coating sequence of the two metal films.

The phenomenon that Ti^4+^ assists and controls X^n+^ diffusion is explained as follows. Ti^4+^ enters into the bulk prior to X^n+^ because of its high mobility. The diffusion of Ti^4+^ into the crystal results in generation of a large number of additional defects, which favors and assists subsequent diffusion of the slower X^n+^ ion. In a sense, Ti^4+^ functions as the path-breaker for X^n+^. One can anticipate that Ti^4+^ can also assist and control the diffusion of other ions that diffuse slower than Ti^4+^. As Ti^4+^ diffuses much faster than X^n+^ and Ti^4+^ diffusion in co-diffusion case is actually in the scenario of single diffusion, it is thus comprehensible that X^n+^ affects Ti^4+^ diffusion little and the Ti^4+^ diffusivity is independent of the thickness of either X^n+^ or the Ti film. The small effect of coating sequence may be explained as follows. Both X^n+^ and Ti^4+^ share the same diffusion reservoir, which is quickly formed in the early stage (*t* < 1 h) of the diffusion procedure^21^,^4^,^5^. The reservoir formed by stacked layers with the Ti film directly in contact with the substrate should have no difference from that with the X^n+^ film directly in contact with the substrate. Thus, the X^n+^ diffusion shows small effect of coating sequence. As for the behavior that different X^n+^ ions gain different extents of diffusion assistance by Ti^4+^, we consider this is associated with the differences in atomic mass, ionic radius and chemical valence as well.

The Ti^4+^/X^n+^ co-diffusion in LN or LT crystal can be modeled by two independent Fick-type equations: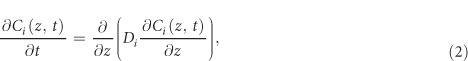 where *C*_i_(*z*, *t*) (*i* = Ti^4+^ or X^n+^) denotes the Ti^4+^ or X^n+^ concentration at depth *z* after diffusion time *t*, and *D*_i_ is the diffusivity of Ti^4+^ or X^n+^. The preceding results and discussion show that the X^n+^ diffusivity is dependent of Ti^4+^-concentration while the Ti^4+^ diffusivity is independent of either the X^n+^- or Ti^4+^-concentration. In addition, *D*_i_ may also depend on the crystal composition. The lower the crystal composition is, the higher the diffusivity of an ion is^19^. Li_2_O out-diffusion phenomenon usually accompanies ion diffusion into the crystal. Regarding the LN and LT samples studied here, it is essential to examine if the phenomenon has taken place in the diffusion procedure. Here, the optical method of refractive index measurement was used to estimate the Li_2_O-content at the doped part of LN or LT sample surface^22^,^23^. A comparison with the initial composition allows us to estimate the extent of Li_2_O out-diffusion. In addition, from the viewpoint of waveguide formation, which is induced mainly by the Ti^4+^ dopants, it is also crucial to know if the X^n+^ diffusion-doping contributes to the refractive index of the LN or LT substrate.

The X^n+^ doping effect on the substrate refractive index can be determined by measuring and comparing the index values at the doped and undoped sample surface parts in the X^n+^-only diffusion case. For the Sc^3+^-, Zr^4+^- and Er^3+^-only diffusion case, the refractive index measurements at the doped and undoped parts of surface show that each dopant has little contribution to the substrate index. This is for both cases of LN and LT. From the measured indices, the Li_2_O-contents at the doped and undoped surface parts of the X^n+^-only doped samples were evaluated on the basis of the Li_2_O-content-dependent Sellmeier equation^22^,^23^. The results show that for the Sc^3+^- and Zr^4+^-only diffusion cases in LN the Li_2_O-content at doped surface equals that at undoped surface and can be thought as identical to that of the as-grown congruent crystal within the experimental error of ±0.1 mol%. This means that Li_2_O out-diffusion is not measurable for the Sc^3+^- or Zr^4+^-only doped LN samples. In the case of Er^3+^-only diffusion in LN, because of the higher diffusion temperature 1130 ^o^C, the 50 h diffusion time resulted in 0.2–0.3 mol% minor Li_2_O content loss due to slight Li_2_O out-diffusion. For the Er^3+^-only diffusion in LT, as the diffusion temperature is below 1400 ^o^C, the Li_2_O out-diffusion resulted in slight Li_2_O content loss of <0.3 mol%. Higher than 1400 ^o^C, Li_2_O out-diffusion caused moderate Li_2_O content loss of <0.6 mol%.

For the case of Ti^4+^/X^n+^ co-diffusion, it is impossible to evaluate the Li_2_O-content accurately at the doped surface because of Ti^4+^ presence, which induces a local index increase. But it is possible to evaluate it indirectly by referencing the situation at the undoped part of the crystal surface. The results show that the Li_2_O-content at the undoped surface part does not reveal a noticeable change from the single diffusion case to the co-diffusion case, and this is the case for all of the co-diffused LN and LT samples under study. Although the situation in the co-diffused layer is sophisticated because of Ti^4+^ presence, the composition at the co-doped surface part should not have a large difference from that at the undoped part.

In words, each X^n+^ dopant has little contribution to the LN or LT index. The index increase in the Ti^4+^/Sc^3+^- or Ti^4+^/Zr^4+^-codoped LN layer is contributed mainly from the Ti^4+^ doping while not from Sc^3+^ or Zr^4+^ doping or Li_2_O out-diffusion. In the Ti^4+^/Er^3+^-codoped LN or LT layer, the index increase includes both the dominant contribution from Ti^4+^ dopants and the slight contribution due to slight Li_2_O out-diffusion. As Li_2_O out-diffusion is not serious for the Ti^4+^/Er^3+^-codoped samples and is not measurable for the Ti/Sc_2_O_3_ and Ti/ZrO_2_ samples, the diffusivities of both Ti^4+^ and X^n+^ can be regarded as Li_2_O-content-independent for all of the LN and LT samples under study. Thus, the Ti^4+^/X^n+^ co-diffusion can be described by two independent Fick-type equations with a constant Ti^4+^ diffusivity and an X^n+^ diffusivity independent of Li_2_O-content but dependent of Ti^4+^-concentration. Whether the diffusivity is constant or concentration-dependent, Eq. (2) has two possible forms of solution: either the erfc or Gaussian function. When the diffusion time *t* is shorter than the depletion time of the diffusion reservoir *t*_1_, the diffusion reservoir is not exhausted and Eq. (2) has an erfc solution. When *t* > *t*_1_, the reservoir is exhausted, the ions diffuse continuously into the bulk, the surface ion concentration degrades and the profile transforms to a Gaussian type. As stated above, the X^n+^ and Ti^4+^ ions in all the studied samples follow either the Gaussian or the erfc profile. Thus, the model proposed is verified by the experiment.

Finally, we would like to give a brief discussion on the potential application of diffusion-controlled feature. Here we exemplify the Ti^4+^/Er^3+^ co-diffusion case in LN. Present study shows that, in the case of co-diffusion with Ti^4+^, the time required to reach a desired Gaussian Er^3+^ profile with a typical 1/e depth of 5–6 μm needs only 50 h due to Ti^4+^-enhanced Er^3+^ diffusion. While in the Er^3+^-only diffusion case, this requires at least 100 h. Therefore, by employing the co-diffusion method the time consumed for Er^3+^ doping and hence the device fabrication period can be shortened substantially. Moreover, as described above, the Ti^4+^ diffusion results in generation of a large number of additional defects, which favors to promote successively the Er^3+^ solubility, Er^3+^ doping concentration and optical gain.

In summary, in the case of single diffusion in LN, Sc^3+^, Zr^4+^ and Er^3+^ ions diffuse respectively five-, four-fold and one order slower than Ti^4+^. In the case of single diffusion in LT, Er^3+^ diffuses at least two orders of magnitude slower than Ti^4+^. In the Ti^4+^/X^n+^ co-diffusion case in LN or LT, the faster Ti^4+^ assists and controls the diffusion of the slower X^n+^ because Ti^4+^ enters into the bulk prior to the X^n+^ due to its higher mobility and the Ti^4+^ diffusion into the crystal results in generation of a large number of additional defects, which favors and assists the subsequent diffusion of the slower X^n+^ ion. The X^n+^ diffusivity can be controlled by the initial thickness of Ti-metal film coated and both follow a linear relationship. Different X^n+^ ions gains different extents of assistance by Ti^4+^. This is associated with the differences of X^n+^ ions in atomic mass, ionic radius and valence as well. The phenomenon that a faster ion assists and controls diffusion of another slower ion should reflect a generalized physical law. Similar phenomenon should also take place in other solids as long as the diffusion of the faster ion can induce generation of defects. This conclusive anticipation needs to be verified in the future by studying more materials such as semiconductor materials. For the diffusion system studied here, the phenomenon can be utilized to largely shorten the device fabrication period, lower the cost, promote the Er^3+^ doping concentration and hence improve the device performance. It can be also utilized to develop new materials.

## Materials and Methods

Commercial Z-cut congruent LN and LT plates with 0.5 mm thickness and optical grade surfaces were used in the present study. These congruent LN and LT plates have the same Li_2_O content of 48.6 ± 0.1 mol%. The ordinary and extraordinary refractive indices at the surface of each as-grown plate were first measured. Stacked Ti (99.99%) metal and Sc_2_O_3_ (99.999%) [or ZrO_2_ (99.99%) or Er metal (99.9%)] films with different thicknesses and coating sequences were then coated onto one surface of each plate, and only half of the surface was coated. The uncoated part is for reference. For the reference too, some LN and LT plates were only coated with Ti, Er, Sc_2_O_3_ or ZrO_2_ film. After the film coating, the plates were annealed in an atmosphere of flowing O_2_ bubbled through room-temperature water at a rate of 1.5 × 10^−3^ m^3^/min. Table 1 summarizes the initial metal and oxide film thicknesses coated onto each LN plate. The LN samples studied can be divided into three groups of Ti/Sc_2_O_3_, Ti/ZrO_2_ and Ti/Er. Each group was subjected to a same annealing procedure. The diffusion temperature/duration was chosen as 1130 ^o^C/50 h for the Ti/Er samples and 1060 ^o^C/10 h for the other samples. The 1060 ^o^C/10 h is the standard fabrication condition for a conventional Ti-diffused waveguide on an LN substrate. Table 2 summarizes the Er and Ti film thicknesses coated onto those LT plates. The LT samples studied here can be classified into two groups, i. e., the Er^3+^-only doped group including samples 1–5 and the Ti^4+^/Er^3+^-codoped group with inclusion of samples 6–12. Different from the LN samples, the LT samples were annealed at different temperatures of 1200–1500 ^o^C and for different durations of 10–30 h. Table 2 specifies the diffusion condition adopted for each sample. Note that the samples in Tables 1 have different initial thicknesses and coating sequences of Ti metal and Er metal or oxide films. The asterisk means that the oxide or metal film was coated first.

After diffusion, the ordinary and extraordinary indices at the doped and/or undoped parts of the surface were measured (at 25 ± 0.1 ^o^C). For the Ti^4+^-only or Ti^4+^/X^n+^-codoped samples, the measurements were carried out only on the undoped surface part because the Ti^4+^-doping induces an increase of refractive index (and hence formation of an optical waveguide). For the X^n+^-only doped samples, the measurements were performed on the X^n+^-doped and undoped surface parts. The refractive index was measured at the 1311 and 1553 nm wavelengths using a commercial Metricon 2010 prism coupler (Metricon Corp., Pennington, NJ), which is based on the working principle of measuring the critical angle of total reflection. The refractive index measured by this method is the value at the crystal surface because the total reflection takes place there. For a Z-cut LN or LT plate, one can choose a transverse magnetic/electric polarization scheme to measure the extraordinary/ordinary index.

Secondary ion mass spectrometry (SIMS) was used to analyze the depth profile of diffused Sc, Zr, Er, Ti ions, as well as the substrate constituents Li, Nb, Ta and O. The analysis was accomplished by a time-of-flight second ion mass spectrometry [ToF SIMS V (ION-TOF GmbH, Münster, Germany)]. A Cs^+^-beam (~45 μm in diameter) with 30–35 nA at 3 keV was used to sputter a crater of either 120 × 120 or 150 × 150 μm^2^ and a pulsed bismuth ion beam of 1 pA at 25 keV was used to analyze the positive or negative secondary ions. Positive secondary ion detection was adopted for the Ti/Sc_2_O_3_ and Ti/Er samples, while negative ion detection was adopted for the Ti/ZrO_2_ samples to improve Zr sensitivity, which is rather low in the case of positive ion detection. Positive or negative ions from a central area of (18.5 × 18.5) - (25.4 × 25.4) μm^2^ inside the crater were detected. During the analysis, a low-energy pulsed electron gun was used to neutralize the positive charges and hence degrade the surface charge accumulation. For the same purpose, before the analysis a 30-nm thick silver film was coated on the sample surface to be analyzed. The trace and the depth of each erosion crater were measured by a Tencor Alpha Step 200 profilometer (KLA-Tencor Corp., Milpitas, CA). The depth resolution was mainly determined by the roughness of crater under analysis and is better than 5 nm in our case.

## Author Contributions

The experiments were conceived, designed and carried out by D.L.Z., Q.Z. and C.X.Q.; Q.Z. and C.X.Q. prepared the samples and carried out the optical measurements; D.L.Z. and D.Y.Y. analyzed and interpreted the data; The SIMS analysis was performed by W.H.W and E.Y.B.P.; D. L. Z. and E.Y.B.P. wrote the manuscript with the assistance of all other co-authors.

## Additional Information

**How to cite this article**: Zhang, D.-L. *et al.* Diffusion control of an ion by another in LiNbO3 and LiTaO3 crystals. *Sci. Rep.* **5**, 10018; doi: 10.1038/srep10018 (2015).

## Figures and Tables

**Figure 1:**
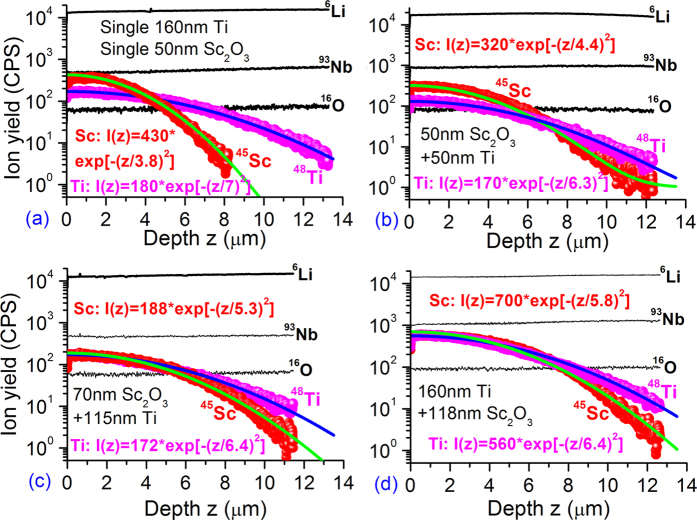
Depth profiles of ^6^Li, ^93^Nb, ^16^O, ^45^Sc and/or ^48^Ti SIMS signals detected from five LN plates coated with Ti and/or Sc_2_O_3_ film.

**Figure 2:**
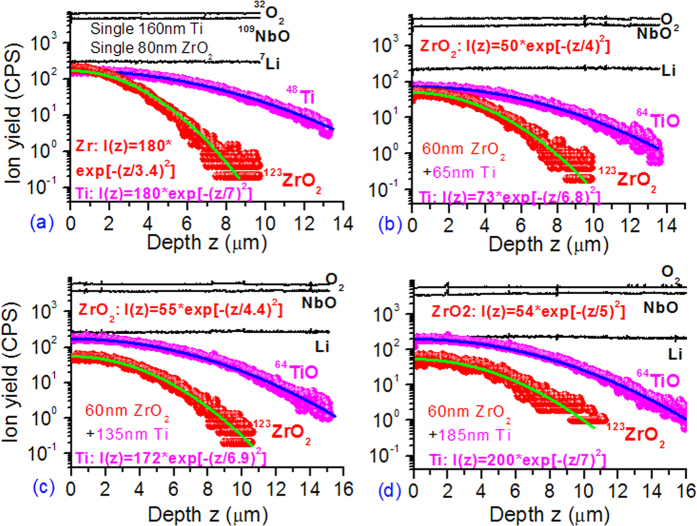
Depth profiles of ^7^Li, ^109^NbO, ^32^O_2_, ^123^ZrO_2_ and ^64^TiO (^48^Ti) SIMS signals detected from five LN plates coated with Ti and/or ZrO_2_ films.

**Figure 3:**
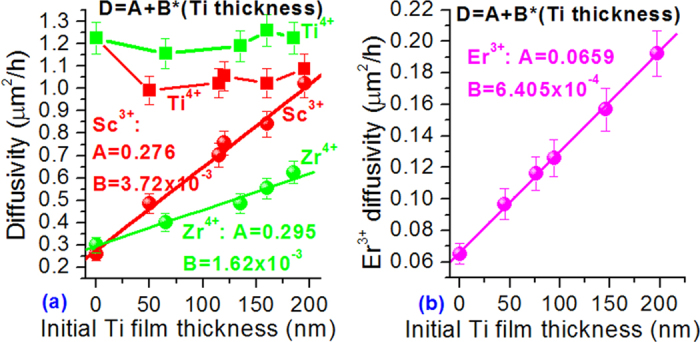
Diffusivity in LN of (**a**) Sc^3+^, Zr^4+^, Ti^4+^ at 1060 ^o^C and (**b**) Er^3+^ at 1130 °C versus initial Ti metal film thickness in the Ti^4+^/X^n+^ co-diffusion case.

**Figure 4:**
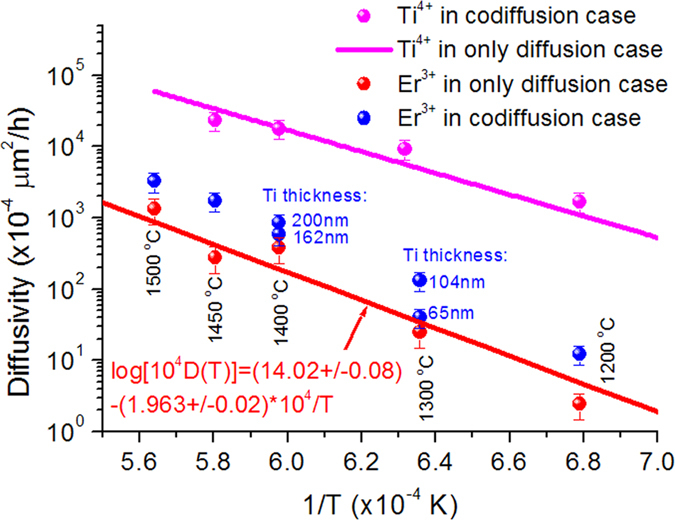
Temperature dependences of Er^3+^ and Ti^4+^ diffusivities in LT. Red balls: Er^3+^ diffusivity in the Er^3+^-only diffusion case; blue balls: Er^3+^ diffusivity in the Ti^4+^/Er^3+^ co-diffusion case; magenta balls: Ti^4+^ diffusivity in the Ti^4+^/Er^3+^ co-diffusion case; magenta line: Ti^4+^ diffusivity in the Ti^4+^-only diffusion case reported in [20]. The red line represents the linear fit to the red balls on the semi-logarithmic scale and the fitting expression is indicated. The blue data indicated near the blue balls denote the initial thicknesses of Ti-metal film coated.

**Table 1:** Summary of initial Sc_2_O_3_/Ti, ZrO_2_/Ti and Er/Ti film thicknesses coated onto LNs.

**Sample No.**	**1**	**2**	**3**	**4**	**5**	**6**	**7**
Sc_2_O_3_/Ti film thickness (nm)	0/160	50/0	50*/50	70*/115	50*/120	118/160*	50*/195
ZrO_2_/Ti film thickness (nm)	—	80/0	60*/65	60*/135	140/160*	60*/185	—
Er/Ti film thickness (nm)	17/0	17*/45	22*/76	22*/94	21*/146	21*/197	22/76*

^*^The oxide or metal film was coated at first.

**Table 2:** Summary of initial Er/Ti film thicknesses coated onto LTs. Also included are the diffusion condition adopted and the Er^3 +^ diffusivity deduced.

**Sample No.**	**1**	**2**		**3**		**4**	**5**
Er/Ti film thickness (nm)	16/0	16/0		12/0		30/0	12/0
Diffusion temperature	1200 °C	1300 °C		1400 °C		1450 °C	1500 °C
Diffusion time	30 h	25 h		17 h		17 h	10 h
Er^3+^ diffusivity (×10^−4^ μm^2^/h)	2.40	25.0		376.5		276.9	1322.5
Sample No.	6	7	8	9	10	11	12
Er/Ti film thickness (nm)	16*/44	25*/65	25*/104	16*/162	16*/200	23/100*	23/160*
Diffusion temperature	1200 °C	1300 °C	1300 °C	1400 °C	1400 °C	1450 °C	1500 °C
Diffusion time	30 h	25 h	25 h	17 h	17 h	13 h	10 h
Er^3+^ diffusivity (×10^−4^ μm^2^/h)	12.1	40.3	130.6	588.2	874.1	1730.8	3240.0

^*^The metal film was coated at first.
